# Metabolomic Prediction of Cadmium Nephrotoxicity in the Snail *Helix aspersa maxima*

**DOI:** 10.3390/metabo14080455

**Published:** 2024-08-17

**Authors:** Aude Devalckeneer, Marion Bouviez, Amandine Gautier, Jean-Marie Colet

**Affiliations:** 1 Laboratory of Human Biology & Toxicology, Faculty of Medicine and Pharmacy, University of Mons, 7000 Mons, Belgium; aude.devalckeneer@umons.ac.be (A.D.);; 2 HEPH (Haute Ecole Provinciale du Hainaut), Condorcet, 7000 Mons, Belgium

**Keywords:** cadmium, nephrotoxicity, invertebrates, ^1^H-NMR metabolomics

## Abstract

The decontamination of polluted soils is a major socioeconomic issue in many industrialized countries. In situ remediation approaches are nowadays preferred to ex situ techniques, but they require among others the use of bioindicators, which are sensitive to the progressive depollution on health effects. Animal species have been mainly used so far to monitor aquatic and air pollution. Current research focuses on the development of living indicators of soil pollution. In this study, the garden snail *Helix aspersa maxima* was acutely exposed to cadmium, one major soil contaminant causing severe health effects, including nephrotoxicity. Kidney and hemolymph were sampled and analyzed by a ^1^H-NMR-based metabonomic approach. Shortly after Cd exposure, numerous metabolic changes occurred in the hemolymph and kidney extracts. Altogether, they were indicative of a switch in energy sources from the Krebs cycle towards b-oxidation and the utilization of stored galactogen polysaccharides. Then, the activation of antioxidant defenses in the renal cells was suggested by the alteration in some precursors of glutathione synthesis, such as glutamate, and by the release of the antioxidant anserin. Cell membrane damage was evidenced by the increased levels of some osmolytes, betaine and putrescine, as well as by a membrane repair mechanism involving choline. Finally, the development of metabolic acidosis was suggested by the elevation in 3-HMG in the hemolymph, and the more pronounced lysine levels were consistent with acute excretion troubles. Cd-induced renal damage was objectified by the increased level of riboflavin, a recognized biomarker of nephrotoxicity.

## 1. Introduction

Past exploitation of soil by coking plants and other petrochemical activity sites has given way today to industrial wastelands that are generally extremely polluted, notably by inorganic elements, including heavy metals such as cadmium. Until recently, the remediation of such polluted soils involved their excavation and remote treatment. In addition to being very expensive, this ex situ approach involves a geographic and temporal movement of pollutants, increasing the risks for the operators and environment due to the possible dispersion of pollutants. In situ remediation approaches were more recently introduced to get rid of the technical, economic and environmental disadvantages mentioned above. Numerous in situ techniques are available, ranging from physical (ventilation, pumping, trapping, etc.) to chemical (washing, oxidation/reduction) to thermal to, last but not least, biological methods (biodegradation, bioventing, biosparging, phytoremediation).

In this context of soil depollution, the physicochemical analysis of soil provides information on the presence and concentrations of anthropogenic pollutants. However, this analysis does not provide any clues on the effects of these contaminants on living systems. Therefore, it is essential to use bioindicators to complement these data when assessing pollution [1,2,3]. These bioindicators can be plant, fungal or animal species present in the contaminated environment (passive use) or established as part of the study (active use). Two types of bioindicators are currently described, namely accumulation indicators and effect or impact indicators. The use of animal bioindicator species has until now been mainly applied to monitoring aquatic pollution (plankton, mussels, “MODIOLE”, “RINBIO” programs) [4,5,6] and air pollution (insects, etc.) [7]. Current research focuses on the development of biological indicators (earthworms, microorganisms, snails) [8] to specifically assess the risk of soil pollution. *Helix aspersa maxima* (Taylor, 1883) [9] is a garden snail of a 20–30 g body weight (b.w.). It originates from North Africa and is considered as a promising alternative model. Terrestrial gastropods are well known for bioaccumulating and neutralizing trace metals either in lysosomal granules and by excretion or by methallothionein binding [10]. The digestive gland of snails has a high affinity for metals due to its ability to absorb particulate materials. The surrounding cells of the digestive gland, including renal cells, are responsible for the absorption, phagocytosis, accumulation and excretion of metals during digestion [11]. Thereby, the kidney is associated to the absorption and urine excretion of these compounds [12].

The biological responses of those bioindicator organisms to toxic exposure result in a change at the biochemical, physiological or behavioral levels. These changes, called toxicity biomarkers [13], can be observable (chlorosis, necrosis, weight loss, loss of pigmentation) and/or measurable (biomass, growth, protein dosage and enzymatic activity) [14,15]. One drawback of this multifactorial approach is the large number of assays that must be carried out to evaluate each individual biomarker. One promising alternative is nowadays proposed by the omics methods, which allows for a more global assessment of the changes in one single analysis. In particular, metabolomics offers a global survey of the alterations in the metabolome, namely, all small organic molecules, players in biochemical and cellular signaling pathways. In this study, we evaluated the potential of combining an invertebrate organism bioindicator of soil pollution with a metabolomics analytical approach to assess Cd-induced nephrotoxicity. To determine this, snails (*Helix aspersa maxima*) were exposed to cadmium (Cd) and we analyzed kidney extracts and hemolymph samples using a ^1^H-NMR -based metabolomic approach.

## 2. Materials and Methods

### 2.1. Animal Housing

All animal experiments were carried out on an invertebrate species, the snail *Helix aspersa maxima*, for which there is no official regulation of animal welfare in Belgium.

*Helix aspersa maxima* individuals were purchased from the helicicole farm of Avesnois (Croix-Caluyau, France) and housed in plastic boxes with permanent access to water and professional food farming provided by the Helinove product (Saint-Paul-en-Pareds, France). They were kept at a temperature of 20 ± 2 °C and daylighting. Only adults with a matching age and body weight (~20 g) were investigated. A total of 24 snails were used in this study, with 6 individuals per group.

### 2.2. Chemicals

Phosphate buffer–D_2_O was prepared with Na_2_HPO_4_.2H_2_O 0.2 M and NaH_2_PO_4_.H_2_O 0.04 M in distilled water–deuterium oxide (D_2_O) solution (80:20, *v*:*v*); methanol; chloroform; TSP (deuterated sodium 3-trimethylsilyl propionate); and CdCl_2_ (cadmium chloride 99.9%, from Alfa Aesar Chemicals, Thermo Fisher Scientific, Waltham, MA, USA).

### 2.3. Cd Exposure

First, the dose of cadmium to which the snails were exposed was selected from the literature data on rodent models [16]. *Helix aspersa maxima* snails (*n* = 12) received an intramuscular injection of 4 mg/kg of Cd. Injections were applied in the posterior part of the foot to minimize the risk of damaging any vital organ of the body with the needle while allowing a systemic distribution of Cd after its absorption in the hemolymph. After either 4 h (*n* = 6) or 24 h (*n* = 6) of exposure, the hemolymph was sampled before killing the individuals by freezing them at −20 °C. Control individuals (*n* = 12) were exposed according to the same protocol to distilled water, solvent used for the preparation of Cd solution. Snails were dissected on ice to sample the digestive gland and kidney tissues, which were immediately frozen in liquid nitrogen and stored at −80 °C until further measurements of enzymatic activity and ^1^H-NMR spectroscopy.

### 2.4. Hemolymph Sampling

The procedure used in this study was based on the validated protocol previously described by Cooper et al. (1993) [17]. Briefly, snails were washed in cold water to remove feces and excess mucus before hemolymph sampling. Snails were placed upside down and a 25 G needle was inserted just below the pneumostome to allow hemolymph to flow out. An amount of 500 µL of a pale blue colored liquid was collected in Eppendorf tubes. Amicon^®^ Ultra-0.5 Centrifugal Filter Devices (Millipore, Merck Group, Darmstadt, Germany) were rinsed four times with 500 μL of demineralized water and centrifuged for 15 min at 14,000× *g*. Then, 500 µL of hemolymph was filtered and centrifuged (30 min at 14,000× *g*) before adding 250 µL of D_2_O and centrifuging again (30 min at 14,000× *g*). Filtrates were kept at −80 °C until ^1^H-NMR analysis.

### 2.5. ^1^H-NMR Spectroscopy and Spectral Data Analysis

The procedure of sample preparation and ^1^H-NMR spectroscopy on tissue extracts used in this study was based on an in-house method developed on *Helix aspersa maxima* tissues [18], derived from the validated protocol previously described by Beckonert et al. (2007) [19].

#### 2.5.1. Sample Preparation

Kidney tissue samples preserved at −80 °C were crushed in a mortar with liquid nitrogen into powder and were extracted with precooled solutions according to a methanol–water–chloroform extraction method (volumes used per gram of organ): homogenization of the formed powder with 4 mL of methanol and 850 µL of water with vortex, followed by 2 mL of chloroform with vortex before extra-homogenization with 2 mL of chloroform and 2 mL of water with vortex. The homogenates were placed on ice during 10 min before centrifugation (15 min at 1000× *g*, 4 °C). The top aqueous phase was retrieved, and methanol was removed in vacuo for 6 h. Each extract was reconstituted in 700 µL phosphate buffer–D_2_O 0.1 M before final centrifugation (10,000× *g*, 10 min), and 50 µL of TSP (D_4_-trimethylsilyl propionic acid) 7 mM was added to 650 µL supernatant. Regarding the hemolymph samples, 600 µL of previously filtered preparations was mixed with 100 µL of TSP 4 mM. TSP prepared in 100% D_2_O was used as the external reference for the calibration of the NMR spectra. Its resonance was arbitrarily fixed at 0.00 ppm for further spectral calibration. Finally, 700 µL of each sample were transferred into individual tubes (5 mm diameter) for NMR analysis.

#### 2.5.2. Acquisition of Metabolic Profiles

One-dimensional NMR spectra of extracts were acquired on a Bruker 500 Avance spectrometer (11.8 T corresponding to a proton Larmor frequency of 500 MHz) at 297 K using a NOESYPRESAT-1d pulse sequence. A total of 128 free induction decays (FIDs), with 65.536 data points per FID, were collected for tissue extraction using a spectral width of 10.330,578 Hz, an acquisition time of 3.17 s and a pulse recycle delay of 3 s. After Fourier transformation, all spectra were referenced to the chemical shift of the TSP (0.0 ppm) and were phase- and baseline-corrected using MestReNova 11.0 software (Mestrelab Research, Santiago de Compostela, Spain) and submitted to a line broadening of 0.3 Hz. After removing spectral regions distorted by imperfect water peak saturation, binning of the remaining areas reducing the spectral region from 0.08 to 10.00 ppm into 248 integrated regions (buckets) with a 0.04 ppm width each was applied. Each spectrum was normalized against the total sum of spectral integrals before multivariate analysis in SIMCA-P+ 12.0 software (Umetrics, Umea, Sweden). An unsupervised principal component analysis (PCA) was used on pareto-scaled data to identify possible outlier samples and evaluate the degree of homogeneity before performing a supervised partial least square discriminant analysis (PLS-DA) to check on the possible clustering of samples by one-way cross-validation analysis of variance (CV-ANOVA) and possibly detect different metabolic patterns due to Cd exposure. The most efficient representation of the groups clustering given by the PLS-DA was the score plot and the corresponding loading plot was used to pick up discriminant metabolites. Metabolites were identified from their respective resonances, according to their chemical shift and multiplicity as compared to historical databases. The quality of the model was described by the R^2^ parameter, which represents the explained variation in the dataset, and the reliability was described by the Q^2^ parameter, which used cross-validation to estimate the goodness of prediction of the model.

#### 2.5.3. Statistical Analysis

Discriminant metabolites were extracted with the variable important parameter (VIP) list of the PLS-DA model, which identified those with a cut-off VIP score ≥ 1 as mainly contributing to the clustering. For ease, the term “area under the curve” (AUC) is used in this work instead of “peak/bucket/feature areas”. A Mann–Whitney Wilcoxon statistical test was applied to the AUC values on those VIPs to assess the significance of metabolites responsible for intergroup differences (*p*-value < 0.05 significance level) between Cd-exposed animals and their matching control fellows (4 h and 24 h). This non-parametric statistical test was chosen considering the semi-quantitative data provided by the AUC values.

## 3. Results

### 3.1. ^1^H-NMR Profiles of Kidney Extracts Collected from Cd-Exposed Snails

**Figure 1:**
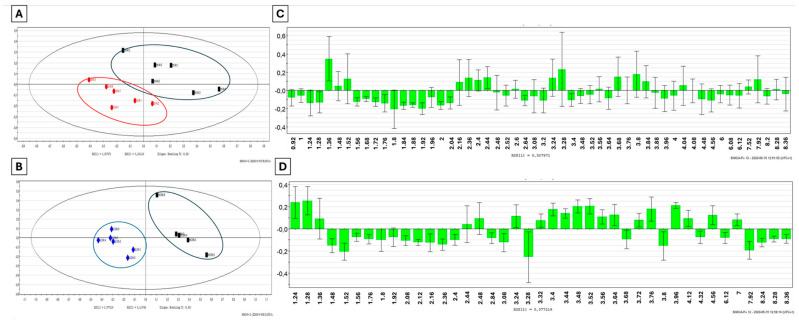
PLS-DA score scatter plots of ^1^H-NMR binned data obtained from kidney aqueous extracts from snails (*n* = 6) exposed to 4 mg/Kg of Cd for (**A**) 4 h (in red) and (**B**) 24 h (in blue) compared to their timely control group (in black). Loading column plots of VIP chemical shifts extracted from the corresponding PLS-DA for Cd exposures (**C**) of 4 h and (**D**) 24 h, respectively.

The ^1^H-NMR spectra obtained from the kidney extracts of the Cd-exposed snails were submitted to multivariate data analyses and compared to the control individuals. The PLS-DA score scatter plot reported in Figure 1 revealed some clusters discriminating those snails exposed to 4 mg/Kg Cd for 4 h from their matched controls (Figure 1A, *n* = 6), with good-quality values of R^2^x = 0.725, R^2^y = 0.998 and Q^2^ = 0.76. The same observation was made between the animals exposed to Cd for 24 h and their respective timely controls with R^2^x = 0.488, R^2^y = 0.963 and Q^2^ = 0.744 (Figure 1C, *n* = 6).

The metabolites contributing to the clustering were identified according to their chemical shift and multiplicity of their corresponding resonances. Peak assignments in the kidney extracts of *Helix aspersa maxima* obtained by ^1^H-NMR were previously described by Devalckeneer et al. (2019) [18] thanks to an in-house library based on the use of Chemonix NMR suite software (version 8.1.1) and the Human Metabolome Database (HMBD). Only those discriminant metabolites displaying a VIP score ≥ 1 were considered for further analysis and are presented in the loading column plots in Figure 1C,D for the 4 h and 24 h time points, respectively.

The changes in the levels of those discriminant metabolites in the renal tissue during Cd exposure are shown in the heatmap projection presented in Table 1: a redder tone indicates a higher renal concentration of the considered metabolite, while a greener tone indicates a lower concentration.

Therefore, increased levels of valine, acetate, N-acetyl-lysine, adenosine and hypoxanthine were observed over time while the levels of lactate and choline decreased. Interestingly, some metabolites only changed after 24 h exposure, such as alanine, aspartate and betaine, which were increased, while riboflavin, β-glucose and glycine were decreased. Many metabolites also varied differently depending on the duration of exposure, namely isoleucine, hydroxybutyrate, fucose, glutamate, citrate, carnitine, glycerol, maltose, β-galactose and anserine, which increased after 4 h and decreased after 24 h compared to glutamine, succinate and urocanate, which followed the opposite evolution. All changes in metabolites following Cd exposure that turned out to be statistically significant according to the Mann–Whitney Wilcoxon test (*p*-value < 0.05) are indicated in bold in Table 1.

### 3.2. ^1^H-NMR Profiles of Hemolymph Samples Collected from Cd-Exposed Snails

**Figure 2:**
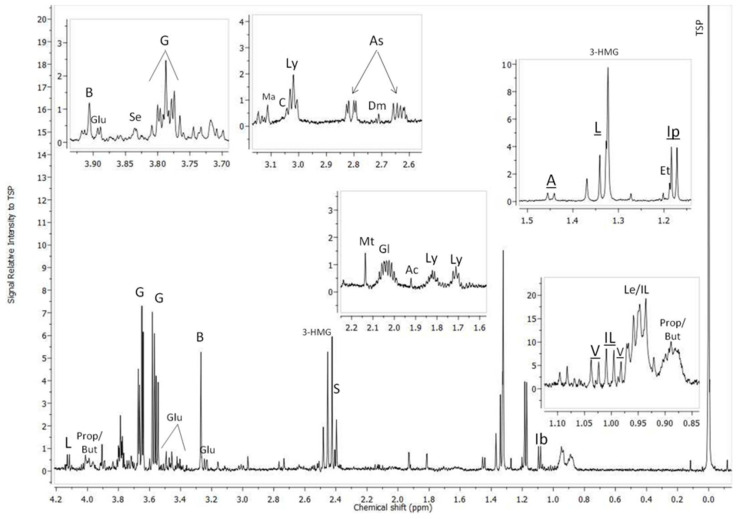
Identification of resonances from ^1^H-NMR spectra acquired at 500 MHz (128 scans) on hemolymph samples from *Helix aspersa maxima*. Peak assignments by comparison of their chemical shift and multiplicity with in-house and web databases (Chenomx NMR suite software, version 8.1.1). V: Valine, Le: Leucine, IL: Isoleucine, P&B: Propanoate/Butanoate regions, Ib: Isobutyrate, Ip: Isopropanol, Et: Ethanol, 3-HMG: 3-hydroxy-methylglutarate, L: Lactate, A: Alanine, Ly: Lysine, Ac: Acetate, Gl: Glutamate, Mt: Methionine, S: Succinate, As: Aspartate, Dm: Dimethylamine, C: Creatine, Ma: Malonate, Glu:Glucose, B: Betaine, G: Glycerol, Se: Serine.

The hemolymph samples were analyzed by ^1^H-NMR spectroscopy and the obtained resonances were assigned to their corresponding metabolites based on their chemical shift and multiplicity by comparison with in-house data and databases freely accessible on the web (Figure 2).

**Figure 3:**
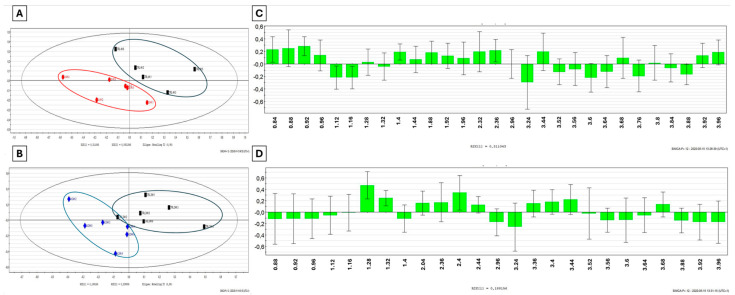
PLS-DA score scatter plots of ^1^H-NMR spectral data obtained from snail hemolymph (*n* = 6) exposed to 4 mg/Kg of Cd for (**A**) 4 h (in red) and (**B**) 24 h (in blue) compared to their timely control group treated with distilled water (in black). Loading column plots of VIP chemical shifts extracted from corresponding PLS-DA for (**C**) 4 h and (**D**) 24 h Cd exposure.

The PLS-DA analysis allowed for the separation of the Cd-exposed group from its matching timely controls, as shown on the score scatter plot in Figure 3. Clusters observed after 4 h Cd exposure (Figure 3A, *n* = 6) showed values of R^2^x = 0.873, R^2^y = 0.996 and Q^2^ = 0.524 and values of R^2^x = 0.92, R^2^y = 0.999 and Q^2^ = 0.689 after 24 h (Figure 3C, *n* = 6), indicating good quality. VIP lists were extracted from each supervised multivariate analysis, pinpointing the discriminant descriptors (Figure 3C,D).

The corresponding identified metabolites listed in Table 2 indicate a timely decrease in the levels of glutamate and glucose associated with increased levels of lysine and betaine. Changes observed after 4 h of exposure, such as increased levels of isobutyrate and isopropanol and decreased levels of acetate, were back to pre-exposure levels after 24 h. Isoleucine, alanine and the butanoate/propanoate region decreased after 4 h of Cd exposure but increased after 24 h. The opposite evolution was noticed for 3-hydroxy-methylglutarate (3-HMG). Changes in metabolites such as lactate, succinate and serine were only seen after 24 h Cd exposure, showing decreased concentrations.

## 4. Discussion

### Metabolic Impact of Cd Exposure in Snails

As most of the basic metabolic pathways are conserved among species through evolution, metabolic biomarkers present great interests in toxicology. Indeed, the enzymes from the glycolytic pathway and Krebs cycle have been identified for a long time in gastropod tissues [20], as well as enzymes involved in the turnover of ketone bodies, which are also detectable in terrestrial mollusks [21].

Shortly after Cd exposure, an increase in the hydroxybutyrate level, a ketone body produced by fatty acid β-oxidation, was noticed in the kidney extracts, together with increased sugar sources such as maltose, fucose and galactose. The higher level of galactose, mainly stored as the polysaccharide galactogen in snails, could indicate a shift towards energy expenditure supported by both β-oxidation and ketogenesis, two major energy sources for renal cells. This increasing glycolytic flux combined with a slowing down of Krebs cycle activity, as witnessed by lower succinate production, were already observed in renal cell carcinoma by Monteiro et al. (2016) [22]. Likewise, an increase in the branched-chain amino acids valine and isoleucine were also observed in our study and by Zhong et al. (2012) [23] in the kidney extracts of rats with chronic kidney disease (CKD). However, the decrease in hydroxybutyrate and carnitine, two main actors of fatty acid metabolism, revealed a dysregulation of β-oxidation after 24 h of Cd exposure. Losses of glucose and other carbohydrates also indicated a lack of primary energy sources. The decreased levels of glutamate and serine in hemolymph confirmed the need for an energy supply, since both metabolites are involved in ATP synthesis. The early glutamate increases in kidney extracts from Cd-exposed snails was most likely correlated with the concomitant decreased levels of urocanate. As serine is a precursor of glycine, a component of glutathione (GSH) with glutamate, one could assume that its consumption supplies the cellular antioxidant defense within 24 h of Cd exposure.

Choline is a methyl donor involved in several physiological processes including the synthesis of phospholipids, essential structural components of cell membranes. The decreased level of choline observed as early as 4 h after the onset of Cd exposure is associated with deviant behavior in cell membrane metabolism. The same metabolic alteration was already reported in CKD patients and explained by a greater demand for phospholipids in replicating renal cells [24]. Choline may also be oxidized in liver and kidney mitochondria to betaine, an osmolyte protecting cell integrity against osmotic stress, especially in the kidney. Elevations in betaine and putrescine, other markers of osmotic stress, were observed in the kidney extracts and hemolymph from 4 h of exposure.

Anserine is a well-known and important buffer molecule in muscles, as well as an antioxidant and crucial promoter of glycolysis [25]. Riboflavin has been described as a biomarker of nephrotoxicity by Fuchs et al. (2011) [26], as well as a key player in muscle repair. Moreover, the metabolic acidosis episode described in CKD rats and leading to the catabolism of muscular proteins can also be appreciated from the higher levels of alanine together with a lowering in glutamate. Thus, the early (4 h) increased anserine level followed by the later (24 h) decreases in riboflavin and glutamate together with an increased alanine concentration could be indicative of the cellular response to Cd-induced renal damage followed by cellular repair, as already reported in rats developing renal failure.

Metabolic acidosis could as well be confirmed by the elevation in 3-HMG in the hemolymph, observed at 4 h exposure. 3-HMG is an intermediate in the leucine degradation process catalyzed by 3-HMG-CoA lyase, also playing a role in the production of ketone bodies. Metabolic acidosis, responsible for the oxidative stress and DNA injuries linked to 3-HMG, was also demonstrated by Deldago et al. (2019) [27].

Purine catabolism was also suspected in the snails during Cd exposure due to higher levels of hypoxanthine and adenosine. Both nitrogen bases can also be related to DNA injuries. Hypoxanthine is generally found in higher concentrations in the plasma of patients with kidney failures. Adenosine, though a nucleoside important in ATP formation, is also a signal molecule known to be induced in the cases of ischemia and hypoxia. Lysine is a major amino acid considered as a building block of most proteins, as well as a precursor of carnitine. Its early increase in the hemolymph of the Cd-exposed snails could be consistent with acute excretion troubles, as already noticed and reported in various types of acute renal failure in the rat [28].

Although this study focused on nephrotoxicity caused by cadmium, it is interesting to note that certain metabolic changes could also reflect nervous system toxicity, another known adverse effect of this heavy metal. Thus, as glutamate and serine are either directly or indirectly involved in neurotransmission, they could be indicative of neurotoxicity. Likewise, as a precursor of acetylcholine and considering the importance of a methyl donor like choline for brain function, decreasing choline could be used to compensate for neuronal injuries. Finally, the accumulation of 3-HMG in biological fluids is a metabolic disorder in patients characterized by a 3-HMG-CoA lyase deficiency and presenting neurological symptoms, especially during metabolic crises [29]. Da Rosa et al. (2013) [30] showed that 3-HMG induces protein oxidative damage in the brain and GSH deprivation leading to a reduction in antioxidant defenses.

## 5. Conclusions

In conclusion, this study demonstrated the potential of combining an “omics” tool with a bioindicator invertebrate model, herein the *Helix aspersa maxima* snail, in prospective risk assessment. This approach could help in monitoring the effectiveness of soil depollution in the future.

## Figures and Tables

**Table 1:** Heatmap of discriminant metabolites in renal tissue with either increased levels (the higher levels in the redder tone) or decreased levels (the lower levels in the greener tone) in Cd-exposed groups as compared to their matching controls. Significant changes are displayed in italic bold character (Mann–Whitney Wilcoxon test, *p*-value < 0.05). Hydroxybut.: Hydroxybutyrate; N-acetyl-LY.: N-acetyl-Lysine; β-glc: β-glucose.

Metabolite	Isoleucine		Valine		Hydroxybut.			Fucose		Lactate
**Control**	100		100		100			100		100
**4 h Cd**	140		120		146			140		33
**24 h Cd**	73		125		***43***			***45***		65
**Metabolite**	**Alanine**		**Putrescine**		**Acetate**			**N-acetyl-LY.**		**Glutamate**
**Control**	100		100		100			100		100
**4 h Cd**	90		***285***		***157***			114		***181***
**24 h Cd**	***208***		108		125			112		83
**Metabolite**	**Glutamine**		**Succinate**		**Riboflavine**			**Citrate**		**Aspartate**
**Control**	100		100		100			100		100
**4 h Cd**	85		80		107			***276***		95
**24 h Cd**	***146***		***162***		63			70		***178***
**Metabolite**	**Choline**		**Betaïne**		**Carnitine**			**β-glc**		**Glycine**
**Control**	100		100		100			100		100
**4 h Cd**	82		90		146			97		96
**24 h Cd**	77		135		***45***			***53***		***63***
**Metabolite**	**Glycerol**		**Maltose**		**β-Galactose**			**Adenosine**		**Anserine**
**Control**	100		100		100			100		100
**4 h Cd**	120		115		201			147		152
**24 h Cd**	***73***		***66***		***44***			***214***		***47***
**Metabolite**	**7.52 ppm**		**Urocanate**		**Hypoxanthine**					
**Control**	100		100		100					
**4 h Cd**	71		80		164					
**24 h Cd**	***52***		***225***		***181***					
***High***	***200***	***185***	***150***	***120***	***100***	***80***	***50***	***25***	***0***	***Low***

**Table 2:** Heatmap of discriminant metabolites in hemolymph with either increased levels (the higher levels in the redder tone) or decreased levels (the lower levels in the greener tone) in Cd-exposed groups as compared to their matching controls. Isoprop.: Isopropanol; Prop/But: Propanoate/Butanoate regions; 3-HMG: 3-hydroxy-methylglutarate.

Metabolite	Isoleucine			Isobutyrate		Isoprop.		Lactate		Alanine
**Control**	100			100		100		100		100
**4 h Cd**	60			134		129		96		69
**24 h Cd**	149			106		101		42		121
**Metabolite**	**Acetate**			**Glutamate**		**Succinate**		**Lysine**		**Glucose**
**Control**	100			100		100		100		100
**4 h Cd**	60			81		100		122		79
**24 h Cd**	110			76		26		132		73
**Metabolite**	**Glycerol**			**Serine**		**Prop/But**		**Betaïne**		**3-HMG**
**Control**	100			100		100		100		100
**4 h Cd**	106			97		73		127		155
**24 h Cd**	112			83		142		133		54
***High***	***200***	***185***	***150***	***120***	***100***	***80***	***50***	***25***	***0***	***Low***

## Data Availability

The original contributions presented in the study are included in the article.
